# A nomogram to predict risk of lymph node metastasis in early gastric cancer

**DOI:** 10.1038/s41598-021-02305-z

**Published:** 2021-11-24

**Authors:** Miaoquan Zhang, Chao Ding, Lin Xu, Shoucheng Feng, Yudong Ling, Jianrong Guo, Yao Liang, Zhiwei Zhou, Yingbo Chen, Haibo Qiu

**Affiliations:** 1 State Key Laboratory of Oncology in South China, Collaborative Innovation Center for Cancer Medicine, Sun Yat-Sen University Cancer Center, Guangzhou, 510060 China; 2 Department of Gastric & Pancreatic Surgery, Cancer Center, Sun Yat-Sen University, Guangzhou, China; 3 School of Public Health (Shenzhen), Sun Yat‐sen University, Guangzhou, 510006 Guangdong Province China; 4 Guangdong Provincial Key Laboratory for Food, Nutrition and Health, Guangzhou, 510080 Guangdong Province China; 5 Guangdong Province Engineering Laboratory for Nutrition Translation, Guangzhou, 510080 Guangdong Province China

**Keywords:** Gastric cancer, Gastrointestinal cancer

## Abstract

Lymph node (LN) metastasis is known as one of the most important prognostic factors for early gastric cancer (EGC) patients. Patients without LNM normally have better prognosis. However, there is no evaluation criteria to accurately assess the possibility of LN metastasis. Therefore, this study aims to establish an effective nomogram for prognosis prediction. In this study, 285 EGC patients from January 2010 to December 2015 were enrolled. Pearson’s Chi-Square (*χ*^2^) test (including continuity correction when appropriate) and logistics regression analyses was used to identify the risk factors for LN metastasis. The independent risk factors identified were then incorporated in a nomogram model. The predictive accuracy and discriminative ability of the nomogram were evaluated by receiver operating characteristic curve (ROC) and calibration curve. LN metastasis occurred in 59 (20.7%) EGC patients. And most of these patients were submucosal cancers (48/59). Chi-square test indicated lymphovascular emboli, carbohydrate antigen 19-9 (CA19-9), ulcer, tumor size, tumor infiltration and histological grade were the risk factors, and multivariate logistics analyses confirmed all these six factors were independent risk factors of LN metastasis, which were selected to construct the nomogram. The nomogram proved well calibrated and had good discriminative ability (C-index value: 0.842). The proposed nomogram could result in more-accurate risk prediction for EGC patients.

## Introduction

Gastric Cancer (GC) is known as one of the most frequently diagnosed cancer and leading cause of cancer deaths in China^1,2^. Early gastric cancer (EGC) is defined as invasive gastric cancer that invades no more deeper than the submucosa, regardless of lymph node (LN) metastasis^3,4^. An overall survival rate (OS) in EGC was considered to be greater than 90% after standardized D2 lymphadenectomy treatment^5^, with LN metastasis being the most significant prognostic factor^6,7^. However, just about 20% of patients exhibited LN metastasis, most of the patients received excessive surgical treatment^8^.

Nowadays, endoscopic resection (ER) has been considered to be an alternative option besides surgery, which could improve the quality of life for patients^9,10^. ER was composed of endoscopic mucosal resection (EMR) and endoscopic submucosal dissection (ESD), with a comparable long-term outcome to surgery^10,11^. However, ER can’t supply treatment of lymph node dissection, so it’s essential to evaluate the status of LN metastasis before and after surgery. According to the Japanese guidelines for ESD and EMR for EGC^12^, the absolute indication of ESD/EMR is as follows: differentiated-type adenocarcinoma without ulcerative findings, of which the depth of invasion is clinically diagnosed as T1a and the diameter is ≤ 20 mm. Only the patients who fulfill the absolute indication are recommended for ER treatment. However, because the absolute indication is excessively strict, the indication for ESD was expanded for the patients with no or lower risks of lymph node metastasis^13^. Therefore, a quantitative prediction model of LN metastasis based on individual information is urgently needed, which is crucial to weigh the benefits and risks of treatment.

A nomogram, also called a nomograph, is a graphical calculating tool, which has been developed in most types of cancer at present^14–16^. However, there are few reports on nomogram of LN metastasis in patients with EGC. In the present study, we aim to establish a nomogram for predicting lymph node metastasis in EGC patients.

## Patients and methods

### Patients

285 EGC patients in total who underwent surgical treatment in Sun Yat-sen University Cancer Center from January 2010 to December 2015 were selected into this retrospective study. The inclusion criteria were as follows: (i) diagnosed as early gastric cancer and (ii) underwent gastrectomy and D2 lymphadenectomy, (iii) achieved radical (R0) resection. The exclusion criteria were as follows: (i) distant metastasis, (ii) multiple cancers, (iii) stump cancer or recurrent cancer, (iv) accepted preoperative treatment, and (v) died in the perioperative period. In addition, those patients with indications of postoperative chemotherapy have accepted chemotherapy, either S-1 or XELOX regimen.

Follow-up assessments including clinical and laboratory examinations, for the first 2 years were conducted every quarterly. During the 3rd to 5th years, assessments were conducted twice a year, and every year after that until death. The primary endpoint was 5-year OS. OS was calculated from the date of surgery until death or to the last follow-up contact, which was used as a measure of prognosis.

### Data collection

We further collected the clinic-pathological data of all included patients. The clinical information included age, gender, surgery type, carcinoembryonic antigen (CEA), carbohydrate antigen 19-9 (CA19-9) and carbohydrate antigen 72-4 (CA72-4) before surgery. The pathological information included depth of tumor infiltration, tumor size, tumor location, Lauren type, ulcer, lymphovascular emboli and histological grade. The depth was measured at the deepest point of penetrated carcinoma cells and tumor size was defined as the maximum diameter^17^. The maximum diameter of the lesions was recorded as the tumor size. The surgery type includes distal, proximal and total resection. The tumor location was separated into three parts (upper third, middle third and lower third part of stomach). Tumor histological grade was divided into two groups: the undifferentiated group, which includes undifferentiated and poorly adenocarcinomas, and the differentiated group, which includes moderately and well differentiated adenocarcinomas^18^.

### Statistical analyses

Statistical analyses to identify risk factors were conducted by the SPSS (version 26.0). The continuous variables were shown as the mean ± standard deviation (SD). The categorical variables were analyzed by Pearson’s Chi-Square (*χ*^2^) test (including continuity correction when appropriate). The independent risk factors of LN metastasis were assessed by multivariate logistic regression analyses. Moreover, the OS were calculated by the Kaplan–Meier method and compared using the log-rank test. Multivariate analyses were used Cox proportional hazards regression models. The primary end point was 5-year OS.

Then, a nomogram was formulated using the package of *rms*^19^ in R studio (version 1.3.10), based on the results of the multivariate logistic regression model. The performance of the nomogram was assessed by calibration curve as indicator of internal calibration, and ROC as a measure of discriminative ability^20^. Meanwhile, the Hosmer–Lemeshow test was used to evaluate goodness of fit of the nomogram.

*P* values (two sided) < 0.05 were considered statistical significance in all statistical analyses.

### Ethical approval statement

All procedures performed in studies involving human participants were in accordance with the ethical standards of our institutional research committee and with the 1964 Helsinki declaration and its later amendments. This article does not contain any studies with animals performed by any of the authors. Due to the retrospective nature of the study, this research was approved by the Ethics Committee of the Sun Yat-sen University Cancer Center, and inform consent was granted a waiver.

## Results

### Clinical-pathological factors and non-parametric test

In total, 285 EGC patients had undergone treatment at our institution from January 2010 to December 2015, who were selected into present study. LN metastasis was confirmed pathologically in 59 (20.7%) of those 285 cases. The average number of retrieved lymph nodes was 32.6 ± 14.5(range 2–98). The median age of those EGC patients was 57 ± 11.5(range, 22–82) years. Non-parametric test indicated that LN metastasis was associated with lymphovascular emboli, CA19-9, ulcer, tumor size, tumor infiltration and histological grade (all *P* < 0.05). Patients with lymphovascular emboli, larger size > 2 cm, or ulcer have a higher possibility of LN metastasis (all *p* < 0.001). Tumors with submucosal invasion was associated with higher LNM metastasis than those with intra-mucosa invasion (*p* = 0.001). Undifferentiated carcinomas were related to lower lymph node metastases (*p* = 0.005). Patients with CA19-9 ≤ 35U/ml have a lower probability of LN metastasis than those with CA19-9 > 35U/ml (*p* = 0.028). But LN metastasis was not correlated with gender, age, CA72-4, CEA, tumor location, surgery type, or Lauren type (Table 1).

**Table 1:** Correlations between LN metastasis and clinic-pathological factors.

	n	LMN		*χ*^2^	*P* value
		Negative	Positive		
All	*285*	226	59		
**Gender**				1.570	0.210
Female	*112*	93	19		
Male	*173*	133	40		
**Age**				1.086	0.297
≤ 65	*222*	179	43		
> 65	*63*	47	16		
**CEA (ng/ml)**				0	1.000*
≤ 5	*265*	210	55		
> 5	*20*	16	4		
**CA72-4 (U/ml)**				0.007	0.779*
≤ 7	*264*	210	54		
> 7	*21*	16	5		
**CA19-9 (U/ml)**				4.820	0.028*
≤ 35	*273*	220	53		
> 35	*12*	6	6		
**Tumor size**				13.458	< 0.001
≤ 2 cm	*107*	97	10		
> 2 cm	*178*	129	49		
**Tumor infiltration**				12.104	0.001
Mucosa	*109*	98	11		
Submucosa	*176*	128	48		
**Lymphovascular emboli**				41.761	< 0.001
Negative	*252*	214	38		
Positive	*33*	12	21		
**Ulcer**				30.176	< 0.001
Negative	*257*	215	42		
Positive	*28*	11	17		
**Histological grade**				7.881	0.005
Differentiated	*113*	99	14		
Undifferentiated	*172*	127	45		
**Surgery type**				2.015	0.365
Distal resection	*239*	189	50		
Proximal resection	*26*	19	7		
Total resection	*20*	18	2		
**Lauren type**				0.634	0.728
Intestinal	*121*	96	25		
Diffuse	*106*	86	20		
Mixed	*58*	44	14		
**Tumor location**				2.456	0.293
Upper third	*46*	40	6		
Middle third	*94*	71	23		
Lower third	*145*	115	30		

*χ^2^ Test with Continuity Correction. *P* < 0.05 indicates statistical significance.

### Multivariate analyses and risk nomogram for LN metastasis

**Figure 1:**
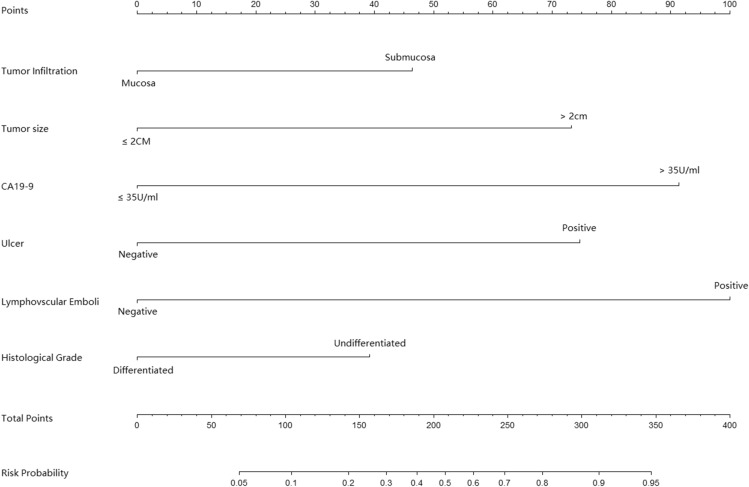
A nomogram predicting the probability of metastatic lymph node involvement for patients with early gastric cancer. The scores of each variable are added to obtain the total score, and then a vertical line is subtracted from the row of total-points to estimate the probability of lymph node metastasis.

**Figure 2:**
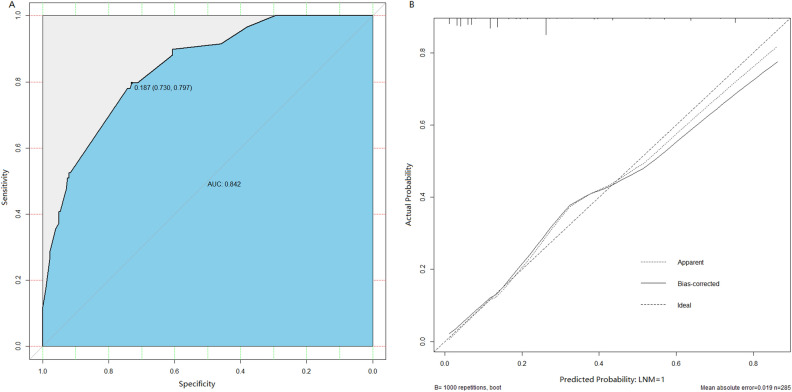
Validation of nomogram for predicting lymph node metastasis in early gastric cancer patients. (**A**) A receiver operating characteristics (ROC) curve of the multivariate logistic regression model. The AUC value was 0.842 (95%CI: 0.795 to 0.882), indicating a good discriminative ability (**B**). Calibration plot. The reference line represents perfect equality of the predicted probability and the actual incidence of lymph node metastasis.

We furthermore summarized both univariate and multivariate logistic analyses of LN metastasis (Table 2). And multivariate analyses confirmed lymphovascular emboli (*p* < 0.001, OR 8.313, 95%CI 3.295–20.973), CA19-9 (*p* = 0.025, OR 6.921, 95%CI 1.316 to 36.401), ulcer (*p* = 0.001, OR 4.861, 95%CI 1.931 to 12.238), tumor size (*p* = 0.001, OR 4.717, 95%CI 1.898 to 11.724), tumor infiltration (*p* = 0.018, OR 2.669, 95%CI 1.176 to 6.061) and histological grade (*p* = 0.035, OR 2.295, 95%CI 1.058 to 4.976) were the risk factors, indicating that all these six factors were independent risk factors of LN metastasis. Therefore, these six clinical-pathological factors were selected to construct a predictive nomogram for LN metastasis (Fig. 1). For each EGC patient, each of these clinicopathological features were assigned points, and the total points was calculated according to the nomogram. The total points corresponded to a probability of predicted LN metastasis. The *p* value of Hosmer–Lemeshow test was 0.287, indicating a good fit of the nomogram. Then a ROC curve was constructed to evaluate the predictive accuracy of the nomogram, which owned an AUC of 0.842 (95% CI 0.795 to 0.882), indicating a good discriminative ability (Fig. 2A). In addition, we developed a calibration curve of the model, which showed that the nomogram performed well with additional 1000 bootstraps (Fig. 2B).

**Table 2:** Univariate and multivariate analysis of LN metastasis risk factors of early gastric cancer.

	Univariate analyse	*P* value	Multivariate analyse	*P* value
	OR (95% CI)		OR (95% CI)	
**Gender**				
Female	1.000			
Male	1.472 (0.802–2.701)	0.212		
**Age**				
≤ 65	1.000			
> 65	1.417 (0.734–2.736)	0.299		
**CEA (ng/ml)**				
≤ 5	1.000			
> 5	0.955 (0.307–2.970)	0.936		
**CA72-4 (U/ml)**				
≤ 7	1.000			
> 7	1.215 (0.424–3.465)	0.715		
**CA19-9 (U/ml)**				
≤ 35	1.000		1.000	
> 35	4.151 (1.287–13.383)	0.017	6.921 (1.316–36.401)	0.022
**Tumor size**				
≤ 2 cm	1.000		1.000	
> 2 cm	3.685 (1.777–7.641)	< 0.001	4.717 (1.898–11.724)	0.001
**Infiltration**				
Mucosa	1.000		1.000	
Submucosa	3.341 (1.649–6.769)	< 0.001	2.669 (1.176–6.061)	0.018
**Lymphovascular Emboli**				
Negative	1.000		1.000	
Positive	9.855 (4.478–21.688)	< 0.001	8.313 (3.295–20.973)	< 0.001
**Ulcer**				
Negative	1.000		1.000	
Positive	7.911 (3.458–18.095)	< 0.001	4.861 (1.931–12.238)	0.001
**Histological grade**				
Differentiated	1.000		1.000	
Undifferentiated	2.506 (1.302–4.823)	0.006	2.295 (1.058–4.976)	0.035
**Surgery type**				
Distant resection	1.000			
Proximal resection	1.393 (0.555–3.498)	0.481		
Total resection	0.420 (0.094–1.871)	0.255		
**Lauren type**				
Intestinal	1.000			
Diffuse	0.893 (0.463–1.721)	0.735		
Mixed	1.222 (0.579–2.574)	0.598		
**Location**				
Upper third	1.000			
Middle third	2.072 (0.779–5.506)	0.144		
Lower third	1.786 (0.692–4.608)	0.231		

*P* < 0.05 indicates statistical significance.

### Comparison of overall survival

**Figure 3:**
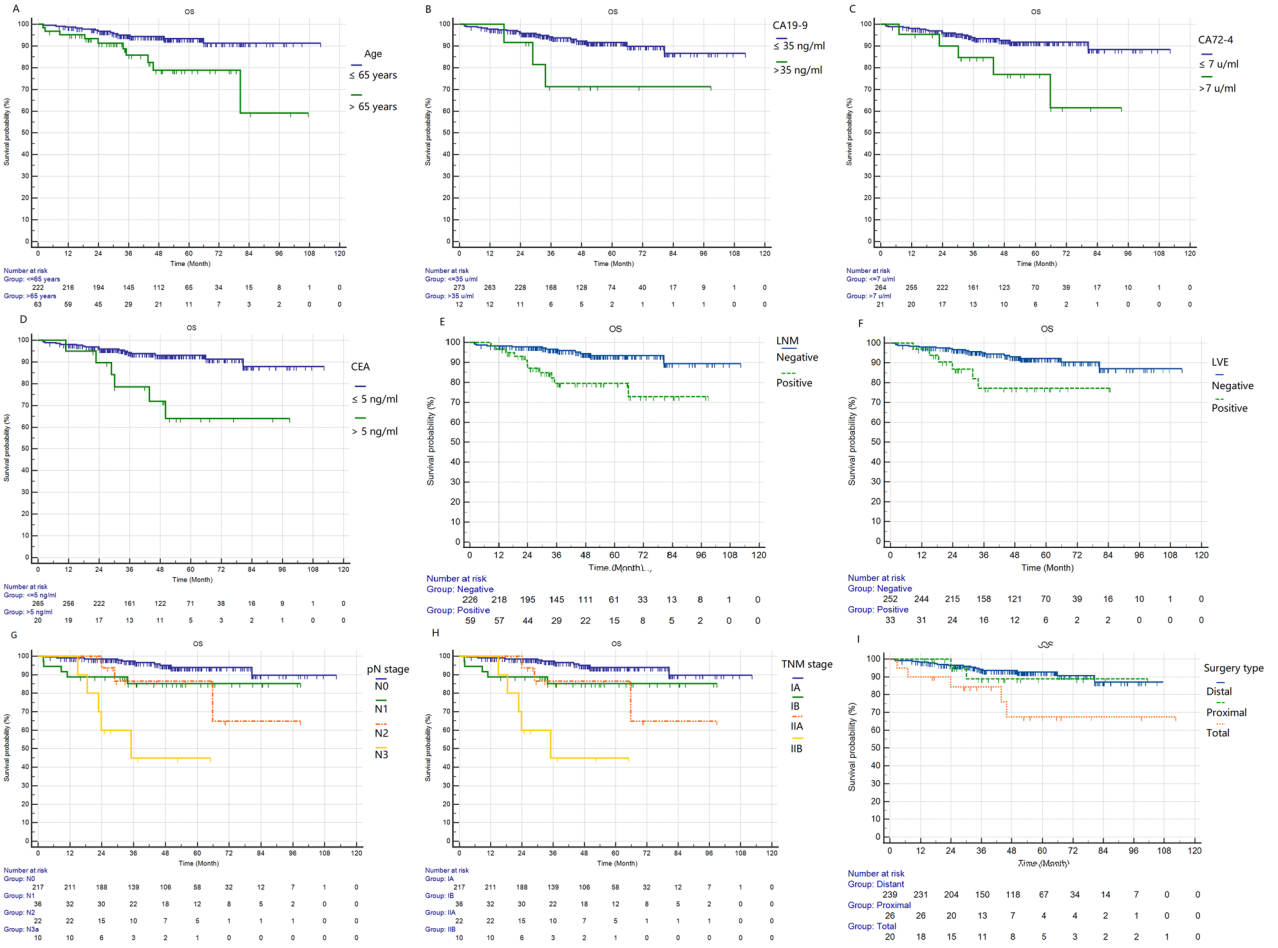
The Kaplan–Meier analyses results of the clinical-pathological features, which proved to be significantly correlated with overall survival. The p values of these features are as follows: (**A**) age, 0.003; (**B**) CA19-9, 0.033; (**C**) CA72-4, 0.009; (**D**) CEA, < 0.001; (**E**) LN metastasis, < 0.001; (**F**) lymphovascular emboli, 0.009; (**G**) pN stage, < 0.001; (**H**) TNM stage, < 0.001; (**I**) surgery type, 0.013.

**Figure 4:**
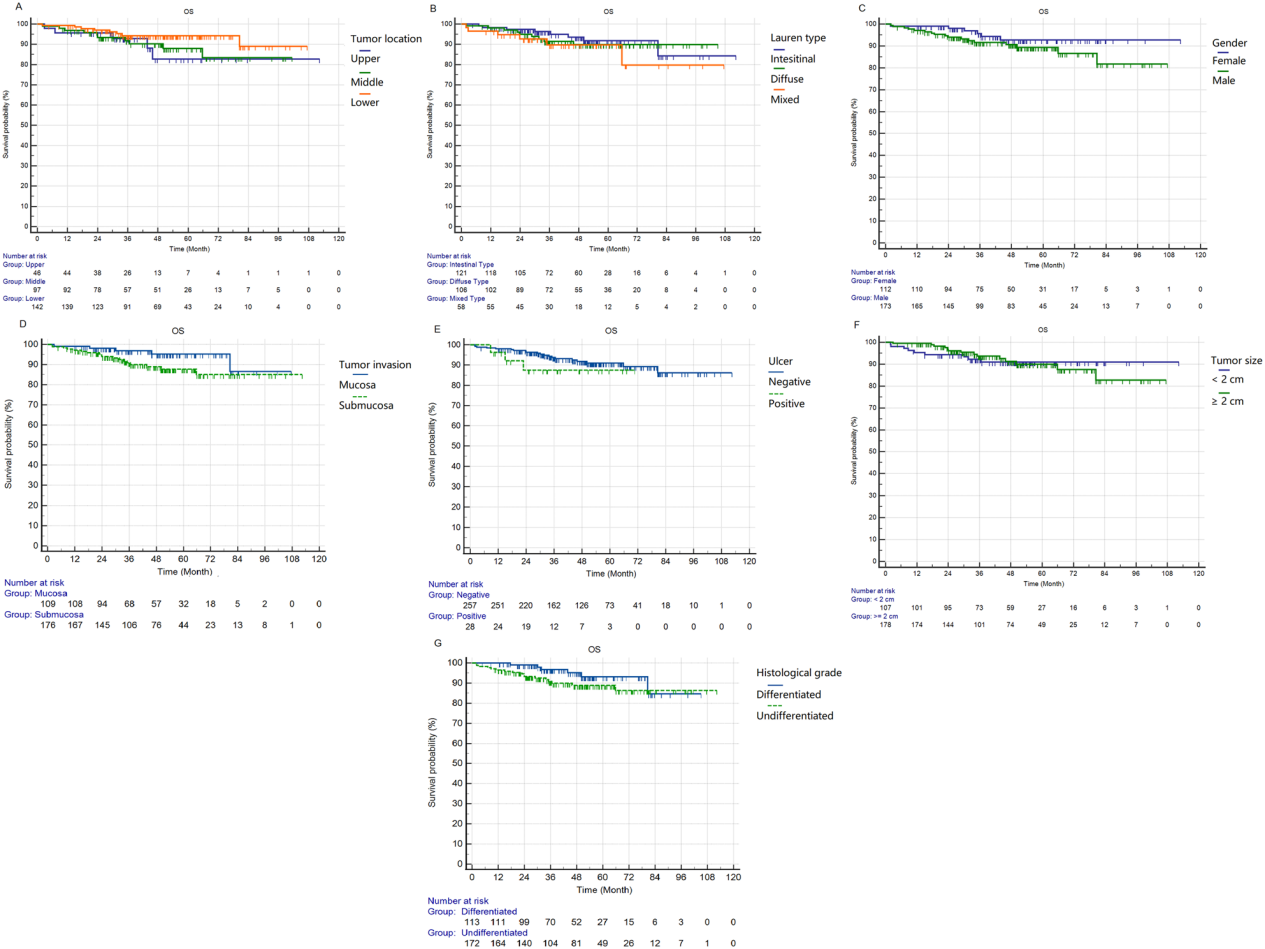
The Kaplan–Meier analyses results of the clinical-pathological features, which showed no significantly correlated with overall survival. The p values of these features are as follows: (**A**) Lauren type, 0.556; (**B**) tumor location, 0.246; (**C**) gender, 0.177; (**D**) tumor infiltration, 0.087; (**E**) ulcer, 0.302; (**F**) tumor size, 0.959; (**G**) histological grade, 0.168.

Of the total 285 EGC patients, the median follow-up time was 46.5 (range 2 -112) mouths. In our study, the 1-year, 3-year and 5-year OS in EGC were 97.9%, 92.6% and 90.6%, respectively, which were calculated by Kaplan–Meier method. Additionally, the 5-year OS of those with LNM and without LNM were 93.4% and 79.5%. The primary end point of this study is 5-year OS. Kaplan–Meier method (Log-rank test) indicated that OS was significantly associated with age (p = 0.003), CEA (p < 0.001), CA19-9 (p = 0.033), CA72-4 (p = 0.009), LN metastasis (p < 0.001), lymphovascular emboli (p = 0.008), surgery type (p = 0.013), pN stage (p < 0.001) and TNM stage (p < 0.001). But it was no relationship with gender (p = 0.177), tumor size (p = 0.959), tumor location (0.246), tumor infiltration (p = 0.324), histological grade (p = 0.168), ulcer (p = 0.302), or Lauren type (p = 0.556). Kaplan–Meier plots are shown in Fig. 3 and Fig. 4. In addition, multivariate Cox regression analyses indicated that only surgery type, CEA and pN stage were independent prognostic predictors (Table 3).

**Table 3:** The result of multivariate Cox proportional-hazards regression.

	Wald *χ*^2^	HR (95.0% *CI*)	*P* value
**CEA**	10.486	4.905 (1.873–12.841)	0.001
**Surgery type**			
Distal resection	7.949	1.000	0.041
Proximal resection	0.623	1.837 (0.406–8.315)	0.430
Total resection	7.786	4.518 (1.566–13.029)	0.005
**pN stage**			
pN0	26.469	1.000	< 0.001
pN1	1.988	1.906 (0.532–6.829)	0.159
pN2	5.269	2.381 (0.590–9.612)	0.022
pN3	25.734	9.166 (2.597–32.353)	< 0.001

*P* < 0.05 indicates statistical significance.

*HR* hazard ratio.

## Discussion

Since long, gastrectomy with D2 lymphadenectomy was considered as the standard and optimal treatment for EGC patients^21–23^. The rate of LN metastasis in patients with EGC ranges from 5 to 22%, which means that approximately 70% to 80% of EGC patients undergo overtreatment with D2 lymphadenectomy^24,25^. The development of less invasive treatments, including endoscopic mucosa resection (EMR) and endoscopic submucosa dissection (ESD), have had an important impact on the treatment strategies revolution in the last few decades^9,10,26^. The EGC patients meeting suitable conditions (low probability of LN metastasis), were recommended to receive less invasive treatments. Therefore, we retrospectively analyzed 285 EGC patients based on clinical-pathological features to identify the risk factors for LN metastasis and construct a nomogram to guide treatment.

In this retrospective study, the incidence rate of LN metastasis was 20.7% in EGC patients. The incidence rate of LN metastasis for the patients with one or more risk features, such as lymphovascular emboli, presence of ulcer, tumor size > 2 cm, submucosa infiltration, undifferentiated type, or CA19-9 > 35 U/ml, was higher than those patients without these clinical-pathological features. Multivariate analyses showed that these six features were independent risk factors for LN metastasis, and the presence of lymphovascular emboli was considered the most important factor. Previous studies have confirmed these features were independent risk factors. Lymphovascular emboli is the characteristic of lymphovascular invasion, which is considered a rate-limiting step in the lymph node metastatic process^27^. And therefore, lymphovascular emboli is significantly associated with LN metastasis^28^. CA19-9 > 35U/ml has been reported as a reliable risk factor for predicting LN metastasis previously^29^. Larger size (≥ 20 mm), presence of ulcer, tumor submucosal infiltration and undifferentiated type were related to regional lymph node metastasis^29–36^. The results of our study were consistent with the studies mentioned above.

And then, we established a nomogram with these six variables related to LN metastasis, which results in more intuitive and accurate in predicting the risk of LN metastasis. The high AUC value (o.842) and calibration curve indicates a good discriminative ability and universal clinical applicability of our nomogram.

LN metastasis has been well considered as one of the most important prognostic factors in both early gastric cancer and advanced gastric cancer^23,37^. For those EGC patients with low probability of LN metastasis, ESD or EMR was the optimal treatment^38^. However, there is still a lack of criteria to evaluate the probability of lymph node metastasis. Our study provides a helpful method to solve this problem. For example, a differentiated mucosal patient without lymphovascular or other features, has a low risk of LN metastasis (less than 5%), and is suitable for EMR. On the contrary, an undifferentiated submucosal patient with lymphovascular emboli, larger tumor size (> 2 cm) and ulcer, has a probability of more than 90% for LN metastasis regardless of other features. This patient is recommended to undergo gastrectomy with D2 lymphadenectomy instead of endoscopic treatment. Thus, we think this quantified and visual predictive model allows clinicians to make more objective decisions on treatment options based on the possibility of LN metastasis.

This study had some potential limitations. Firstly, the final selected sample size is small, so we consider improving the nomogram by expanding the sample size in the future. Secondly, this was a single-center retrospective study. In order to further improve the clinical application environment of the nomogram, additional external validation on different populations is required. Finally, P53, HER-2 or other genetic information was not included in the nomogram, which may provide more individualized evidence of our nomogram. In the future, we intend to incorporate more variables into the nomogram to improve its accuracy, including the information of imaging examination and molecular detection. In spite of the above limitations, we believe that our rosette map can be helpful for clinical work. A strength of our nomogram is that it was built from the variables which could be easily obtained before ESD or EMR treatment. It therefore means that this nomogram can be used to preliminarily determine the risk of lymph node metastasis of a patient before surgery, and hence to prescribe a preliminary treatment.

## Conclusion

In conclusion, we established a nomogram to help clinicians predict the risk of lymph node metastasis in the patients with early gastric cancer. This tool could help clinicians and patients quantify the potential incidence of lymph node metastasis. Surgery plus lymph node dissection is recommended for certain patients with a high risk of LN metastasis, and endoscopic dissection is suitable for those with a low risk. In the future, we will conduct further research to improve clinical application value of the nomogram.
